# Non-contiguous three-level hybrid surgery with C2-3 cervical disc arthroplasty: a case report and literature review

**DOI:** 10.3389/fsurg.2025.1706862

**Published:** 2025-12-02

**Authors:** Xiaoqiang Zhao, Yaling Li, Shihao Chen, Minghe Yao, Yi Deng, Tingkui Wu, Kangkang Huang, Beiyu Wang

**Affiliations:** 1 Department of Orthopedic Surgery, Sichuan University West China Hospital, Chengdu, China; 2 West China Hospital School of Nursing, Sichuan University, Chengdu, China; 3 Operating Room, Department of Anesthesiology, West China Hospital, Sichuan University, Chengdu, China

**Keywords:** hybrid surgery (HS), cervical disc arthroplasty (CDA), anterior cervical discectomy and fusion (ACDF), C2-3, non-contiguous three-level

## Abstract

**Background:**

Cervical disc herniation at the C2-3 level, resulting in cervical spondylotic myelopathy (CSM), is an uncommon clinical entity. The diagnostic and therapeutic complexity escalates when this pathology coexists with non-contiguous multilevel cervical disc degenerative disease (CDDD). Due to the segmental variability in pathological features, the clinical manifestations of such cases are highly heterogeneous, thereby necessitating a highly individualized treatment strategy. Hybrid surgery (HS), which integrates cervical disc arthroplasty (CDA) and anterior cervical discectomy and fusion (ACDF), offers a tailored approach for the management of multilevel degenerative cervical pathology. The unique anatomical features and surgical technical challenges at the C2-3 level impose significant constraints on treatment options. This article presents a case of non-contiguous three-level hybrid surgery involving CDA at C2-3 and ACDF at C4-5 and C5-6, and discusses the feasibility of this technique for upper cervical disc pathology.

**Case presentation:**

A 62-year-old female was admitted with a 6-month history of neck and right upper limb pain, numbness, and gait instability, which had been unresponsive to conservative management. DR revealed loss of the normal cervical lordosis. CT showed no significant osteophyte formation or bony canal stenosis. MRI demonstrated a large disc extrusion at C2-3 causing spinal cord compression, and disc herniations at C4-5 and C5-6 with nerve root impingement. Based on clinical and imaging findings, a diagnosis of multilevel cervical spondylopathy (C2-3, C4-5, and C5-6 disc herniation) was established. The patient underwent anterior cervical discectomy followed by artificial disc arthroplasty (CDA) at C2-3, and anterior cervical discectomy and fusion (ACDF) at C4-5 and C5-6, successfully completing a non-contiguous three-level hybrid surgical procedure.

**Results:**

Postoperative symptoms were significantly alleviated. At the 12-month follow-up, pain and gait disturbance had largely returned to normal. MRI confirmed adequate decompression of neural compression, DR demonstrated satisfactory range of motion (ROM) at C2-3, and CT revealed satisfactory bone healing at the fused segments.

**Conclusion:**

CDA serves as an effective alternative for C2-3 disc pathology, achieving neural decompression while preserving segmental mobility. The HS provides a valuable surgical option for the precise treatment of non-contiguous multilevel degenerative disease.

## Introduction

Hybrid surgery (HS), a combination of anterior cervical discectomy and fusion (ACDF) and cervical disc arthroplasty (CDA), is applicable for the treatment of both single-level and multi-level cervical degenerative disc disease (CDDD). Non-contiguous CDDD is a special form of multi-level CDDD. As a clinically rare pathological condition, the degeneration of the cervical discs in this disease is non-contiguous, with one or more normal discs between two or more degenerated cervical discs. The therapeutic effect of HS for the treatment of multi-level CDDD has been proven to be non-inferior to ACDF or CDA alone (1, 2). Due to the presence of uncontiguous lesions, managing the intermediate normal segments poses a challenge in the treatment of this type of cervical spondylosis, where personalized management for each disc segment level is particularly important (3). According to the results of a finite element study, HS has the biomechanical advantage in the treatment of non-contiguous CDD for potentially reducing the likelihood of loss of intervertebral disc height (IDH) of the intermediate segment (IS) between CDA and ACDF (4, 5). More importantly, CDA demonstrates significant biomechanical advantages by effectively mitigating stress concentration in intermediate and adjacent spinal segments (6).

Compared to the common degenerative changes in the lower cervical spine, C2-3 disc herniation is much rarer. The literature mentions that C2-3 pathologies are usually associated with trauma or hangman's fractures (7). Surgical options for C2-3 disc herniation include anterior decompression and fusion, posterior decompression and fixation, as well as other methods. Compared to posterior cervical surgery, C2-3 anterior surgery has advantages such as fewer complications and less postoperative axial neck pain, although the surgical approach can be challenging (8–10). Reported outcomes of C2-3 ACDF have shown satisfactory clinical results and are often recommended for unstable hangman's fractures (11, 12). Owing to the limitations of ACDF surgery, such as the loss of mobility in the operated segment and the increased risk of ASD, CDA offers more advantages by preserving mobility and avoiding stress concentration in the intermediate and adjacent segments. Additionally, the range of motion (ROM) of C2-3 segment plays a crucial role in maintaining cervical stability, making CDA a more suitable option (6, 13). Therefore, in our report, we adopted a HS approach to provide individualized treatment for each segment of non-contiguous cervical lesions, aiming to maintain cervical stability and protect the intermediate segments as much as possible.

## Case presentation

### Preoperative assessment

**Figure 1:**
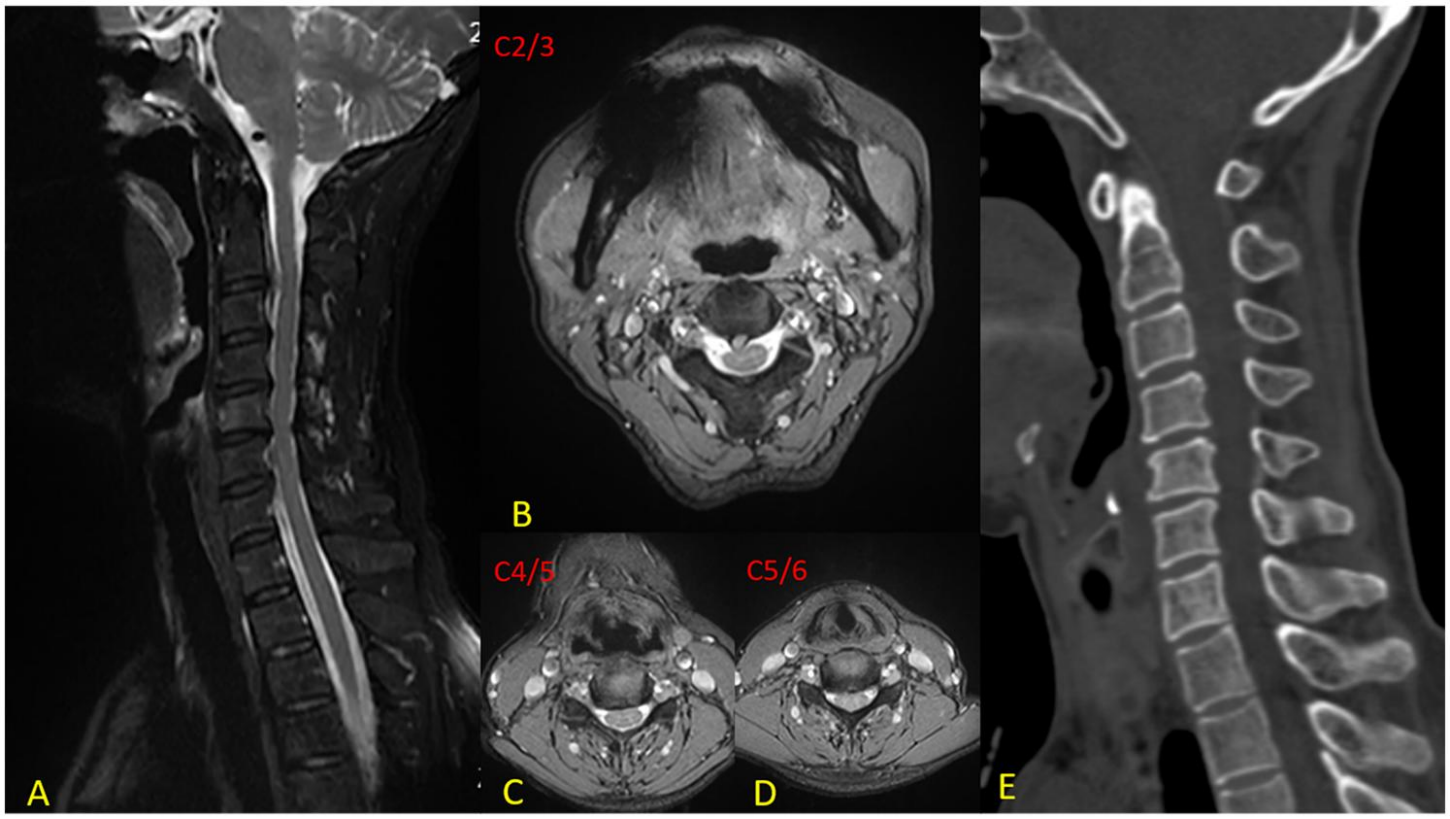
Preoperative imaging. **(A)** Sagittal MRI reveals a massive disc extrusion at C2-3 resulting in secondary spinal canal stenosis, along with disc protrusions at C4-5 and C5-6; **(B)** Axial T2-weighted image at C2–3 demonstrates a large right paramedian soft disc extrusion; **(C)** Axial view at C4–5 shows a disc protrusion causing neural compression; **(D)** Axial image at C5–6 illustrates disc herniation leading to neuroforaminal stenosis; **(E)** CT scan depicts osteophyte formation surrounding the C4–6 vertebral bodies, ruling out ossification of the posterior longitudinal ligament (OPLL).

The patient is a 62-year-old woman who presented with gait instability and recurrent pain and numbness in the neck, shoulder, and right upper limb for 6 months [Visual Analogue Scale (VAS) score = 7, Japanese Orthopaedic Association score (JOA) = 10, Neck Disability Index (NDI) = 23]. The pain was characterized by a stabbing sensation radiating to the thumb, index, and middle fingers. Symptoms were exacerbated by coughing, sneezing, or physical exertion. Motor examination showed 4/5 strength in the right deltoid, biceps, and triceps muscles, with 5/5 strength in the remaining limb muscles. Hoffmann's sign was positive while Babinski's sign was negative. She underwent muscle relaxation exercises and medical treatment for over 6 months without improvement, and the pain and numbness persisted. Preoperative MRI revealed degenerative changes and extrusion of the C2-3 disc with secondary spinal canal stenosis; disc herniations at C4-5 and C5-6 caused nerve compression. CT scans ruled out cervical ossification of posterior longitudinal ligament (OPLL) (Figure 1). Additionally, lateral bending and extension dynamic x-rays successfully demonstrated good cervical spine mobility from C2 to C7. The preoperative ROM at C2-3, C4-5, and C5-6 are 6.7°, 8.8°, and 8.8°, respectively. The patient had limited neck movement and complained of neck stiffness. The bone mineral density (BMD) report indicated a T-score of −0.8. Electromyography (EMG) findings revealed neurogenic injury in the right biceps and triceps muscles. Based on the aforementioned clinical manifestations and preoperative examination results, we devised an individualized surgical strategy for the patient, taking into account the existing anterior nerve compression, discontinuous disc pathology, and the unique feature of a normal C3-4 segment. Specifically, we performed CDA at the C2-3 level and ACDF at the C4-5 and C5-6 levels.

### Surgical procedure

**Figure 2:**
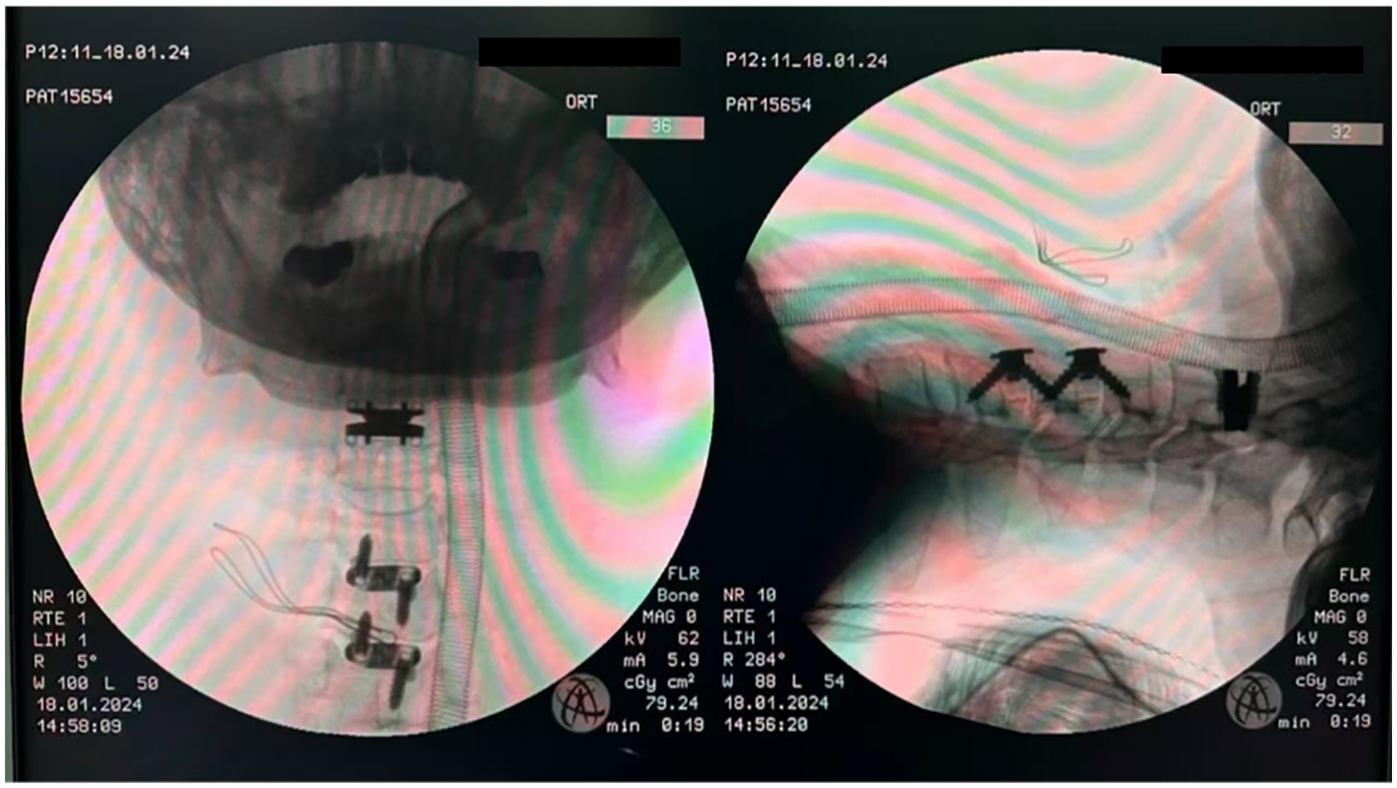
Intraoperative use of C-arm fluoroscopy to confirm the correct placement of the implant.

During the surgery, after general anesthesia induction and endotracheal intubation with laryngoscopic assistance, the patient was maintained in a neutral position (with mild cervical extension). A standard right anterior cervical approach and exposure were performed. A transverse incision approximately 7 cm in length was made in the anterior neck, and the approach was through the interval between the carotid sheath and the tracheoesophageal sheath. Initially, the surgeon completely resected the intervertebral disc tissue at the corresponding level. Bilateral uncovertebral joints and osteophytes were removed using a high-speed drill or Kerrison rongeurs to achieve thorough decompression until pulsation of the dural sac was visible. Secondly, for CDA, after preparing the endplates and performing an insertion trial, an appropriately sized Prestige-LP (Medtronic Sofamor Danek, Memphis, TN) was inserted along with the channels in the endplates. Thirdly, for fusion, after determining the appropriate size of the trajectory spacer, the corresponding Zero-P implant (Synthes, Oberdorf, Switzerland) filled with β-tricalcium phosphate or locally harvested bone was inserted into the prepared intervertebral space. Next, two locking screws were tightened cranially and caudally to secure the implant. Then, intraoperative C-arm fluoroscopy was used to confirm the correct placement of the implants (Figure 2). The natural structure of the intervertebral space and prevertebral tissues were preserved during the surgery. Finally, a drain was inserted before closing the incision. After the patient was fully awake, following commands, and had regular and strong spontaneous breathing with satisfactory tidal volume, the endotracheal tube was removed. The patient was closely monitored in the ICU for 24 h. In the absence of dyspnea, stridor, or decreased oxygen saturation, the patient was transferred to a general ward for further observation.

### Postoperative course

**Figure 3:**
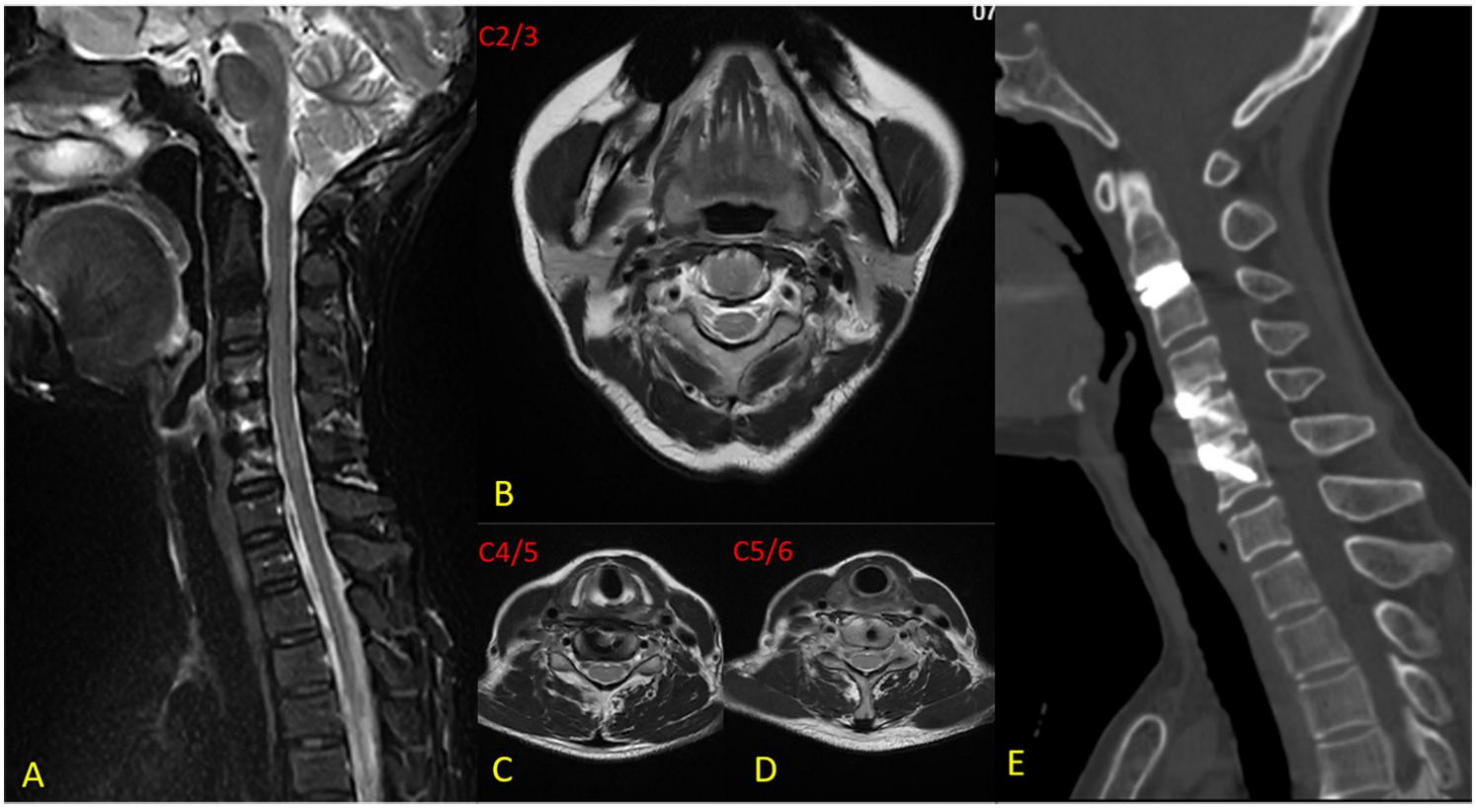
Imaging at 12-month follow-up. **(A)** Sagittal metal artifact-reduced MRI demonstrates complete resolution of neural compression at C2-3, C4-5, and C5-6 levels, with no residual occupying lesion in the spinal canal; **(B)** Axial view at C2-3 confirms thorough neural decompression without residual compression; **(C)** Axial image at C4-5 shows adequate decompression of previously compressed neural structures; **(D)** Axial image at C5-6 illustrates complete relief of spinal cord and nerve root compression; **(E)** CT scan reveals well-positioned artificial cervical disc at C2-3 and Zero-P implants at C4-5 and C5-6, with satisfactory bony fusion.

**Figure 4:**
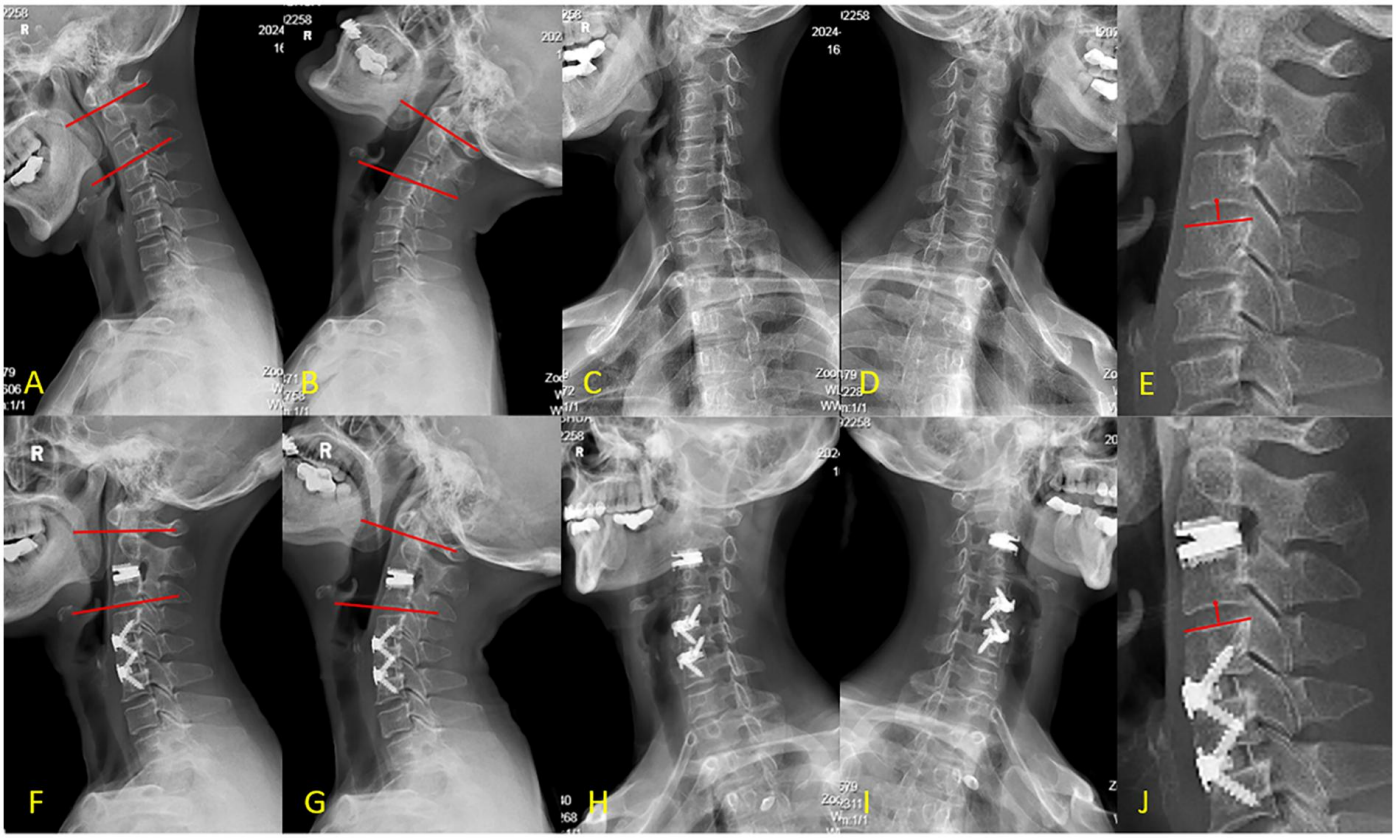
**(A–E)** Preoperative x-rays, F-J: postoperative x-rays. Image **(A)** and **(B)** shows preoperative C2-3 range of motion was 7.1°. Image **(F)** and **(G)** shows postoperative range of motion was 6.9°. Image **(C)** shows stenosis of the C4-5 and C5-6 intervertebral foramina. Image **(H)** shows complete resolution of the stenosis with the intervertebral foramina enlarged compared to preoperative status. Image **(E)** shows preoperative intervertebral disc height (IDH) of C3-4 is 14.1 mm, Image **(J)** shows postoperative IDH of 13.4 mm with no loss.

The patient's hospital course was uneventful, with no occurrence of C5 palsy, hematoma, dysphagia, hoarseness, or dysphonia. Significant symptomatic relief was noted immediately after surgery (postoperative VAS score = 2), although intermittent neck, shoulder, and right upper arm pain persisted. Gait instability had largely resolved. Postoperative MRI confirmed complete decompression of neural compression and CT revealed satisfactory bone healing at the fused segments (Figure 3). At the 12-month follow-up, dynamic radiographs (including flexion–extension lateral views) demonstrated preserved ROM at each surgical level: preoperative ROM measured 6.7°, 8.8°, and 8.8° at C2-3, C4-5, and C5-6, respectively, compared to 6.0°, 9.5°, and 8.4° postoperatively. Notably, no loss of IDH was observed at the IS (C3-4), and its mobility remained unchanged (Figure 4). The patient reported only mild pain (VAS = 2), with complete resolution of preoperative gait instability and persistent discomfort in the neck and shoulder [JOA = 16, NDI = 5]. No postoperative complications were noted during the follow-up period, and cervical spine mobility was well maintained.

## Discussion

There are multiple surgical options for the treatment of C2-3 cervical disc herniation. For upper cervical lesions, posterior surgery is a safe choice (9). However, compared to posterior cervical surgery, the anterior approach has advantages such as fewer complications and less postoperative axial neck pain (8). Anterior cervical surgery includes different surgical approaches, such as the standard right anterior cervical approach, transfacetal approach, transoral approach, anterolateral epidural approach, and submandibular approach (8, 14–17). Nevertheless, C2-3 surgery is particularly challenging due to the complex surrounding anatomy (18). The C2-3 surgical procedure is particularly challenging due to the complex surrounding anatomical structures. Specifically, the C2-3 segment is in close proximity to the medulla oblongata and upper cervical spinal cord, while also adjacent to the vertebral artery and internal carotid artery (19). These spatial constraints significantly limit the operating space, making intraoperative precise decompression and fixation extremely technically demanding. In addition, the atlas and the second cervical vertebra form the atlanto-axial joint, which constitutes the main structure for head rotation. During surgery to thoroughly decompress and relieve nerve compression, imprecise resection may disrupt spinal stability, and extensive fixation and fusion may lead to limited cervical spine mobility. Therefore, when operating in the C2-3 region, it is necessary to balance decompression and cervical stability, requiring high precision to maintain joint stability and avoid postoperative cervical instability. To date, there is no unified consensus on the treatment of C2-3 cervical disc herniation. Previous retrospective studies have shown that ACDF is a widely used surgical method for C2-3 cervical disc herniation (12). As an alternative to ACDF, this case report demonstrates that in addition to fusion, CDA can also be performed effectively and safely.

Considering the anterior neural compression, discontinuous and variable disc degeneration, and normal C3-4 segment in this case, an individualized surgical strategy was devised to achieve complete neural decompression, preserve the C3-4 disc, and maintain cervical spine mobility. Anterior surgery was preferred over posterior approaches due to its direct access to anterior neural compression and ability to restore intervertebral height and stability with implants. Given the anterior osteophytes at C4-5, significant posterior endplate sclerosis at C5-6, and substantial height discrepancy in the intervertebral space, ACDF was selected for C4-5 and C5-6 to restore height and lordosis and achieve solid fusion. For the large central disc herniation at C2-3, surgical intervention was necessary to relieve neural compression. Moreover, the absence of significant disc degeneration and spinal cord compression at the C3-4 level, along with the need to preserve the rotational stability of the atlantoaxial joint by avoiding excessive restriction of C2 vertebral motion, and to prevent stress concentration and subsequent ASD at C3-4, made CDA at C2-3 a more suitable option. Importantly, the C2-3 level had favorable bone quality, consistent intervertebral height, and no significant osteophytes or endplate sclerosis, supporting CDA as the optimal surgical strategy. Therefore, we performed a hybrid surgery combining CDA at C2-3 with ACDF at C4-5 and C5-6, achieving an individualized, non-contiguous three-level procedure.

ACDF recognized globally as the “gold standard” for anterior cervical surgery, is characterized by its broad indications, mature technique, high fusion rate, and strong stability (20). Over the past two decades, CDA has emerged as an established alternative to ACDF for the treatment of radiculopathy and myelopathy, favored by spine surgeons for its ability to maintain motion at the treated segment, potentially reduce adjacent segment degeneration, and demonstrate satisfactory long-term clinical outcomes with low surgical complication and revision rates in long-term follow-up (20, 21). As a combination of ACDF and CDA, HS encompasses the advantages of anterior cervical surgery. Depending on the location of the lesion, different combinations are selected after precise identification of indications to provide the optimal treatment strategy for different lesion levels (2). ACDF fuses and fixes adjacent vertebrae, which may lead to increased stress on adjacent segments and accelerate their degeneration. Studies have shown that compared to ACDF, HS has a smaller mechanical impact on adjacent segments and can reduce the interaction between CDA segments and ACDF segments (1, 22). This makes HS a suitable option for the treatment of non-contiguous CDDD (23).

Despite the advantages of HS in preserving cervical spine mobility and reducing the incidence of ASD, surgeons must be vigilant about potential risks such as periprosthetic bone absorption and heterotopic ossification (HO) (24). Anterior bone loss (ABL) of vertebra is a common early postoperative complication, which is usually self-limiting and occurs within the first year after surgery. Wu et al. (25) reported an incidence of ABL of 52.8% in patients who underwent CDA with the Prestige-LP prosthesis. Severe ABL can lead to adverse outcomes such as implant subsidence and displacement. Fortunately, our follow-up results did not reveal any significant bone absorption around the C2-3 CDA. Moreover, HO is another potential long-term complication of CDA. Clinical series comparing ACDF with CDA indicate that HO remains an important factor affecting the long-term success of motion-preserving implants (26). Although CDA is designed to preserve motion, severe HO can restrict segmental mobility, and in some cases, lead to complete loss of segmental motion, thereby threatening the longevity of the cervical prosthesis. It can also negatively impact surgical outcomes, causing postoperative neck pain, stiffness, and restricted mobility. Further research and longer-term follow-up are still needed to support the use of CDA at the C2-3 level.

We evaluated published English literature by searching EMBASE, PubMed, Medline, and Scopus. To date, only two cases of CDA at the C2-3 level have been reported. One such case, as described by Yang et al. (27), involved a 49-year-old female patient who successfully underwent non-contiguous two-level CDA at C2-3 and C5-6 using Prestige-LP artificial discs under nasal anesthesia intubation. During the 2-year postoperative follow-up period, this procedure achieved relatively satisfactory clinical outcomes. Another case involved Jason Ku et al. (28) performing contiguous three-level CDA from C2-5 using Mobi-C (Zimmer-Biomet) artificial discs in a 52-year-old male patient. Postoperatively, the range of motion at each surgical level was preserved, with satisfactory outcomes observed during follow-up. At present, it remains uncertain whether C2-3 level CDA can be applied to non-contiguous CDDD.

In this case report, we demonstrate the successful implementation of standard anterior cervical CDA at C2-3 despite the high technical challenges. Furthermore, the non-contiguous three level hybrid surgical approach combining C2-3 CDA with C4-5 and C5-6 ACDF achieved successful clinical outcomes in this patient. During the 12-month follow-up period after the surgery, the clinical and radiographic results of this case are good. Although CDA has not yet been universally accepted as the standard treatment for cervical disc herniation at the C2-3 level, it appears to represent a safe and feasible surgical option. For patients presenting with isolated C2-3 disc herniation who maintain normal neurological function and cervical lordosis, the strategic use of CDA to preserve segmental mobility is clinically justified and merits particular attention in the field of spinal surgery.

## Conclusion

This case report demonstrates that CDA represents a viable alternative for managing degenerative disc disease at the C2-3 level, providing satisfactory clinical outcomes while preserving segmental mobility. This approach broadens the therapeutic spectrum beyond fusion-based techniques for high cervical disc pathology. Furthermore, the hybrid surgical strategy offers a tailored treatment option for non-contiguous cervical disc disease, enabling precise and level-specific surgical intervention.

## Data Availability

The original contributions presented in the study are included in the article/Supplementary Material, further inquiries can be directed to the corresponding author.
